# MicroRNA expression patterns in canine mammary cancer show significant differences between metastatic and non-metastatic tumours

**DOI:** 10.1186/s12885-017-3751-1

**Published:** 2017-11-07

**Authors:** Malgorzata Bulkowska, Agata Rybicka, Kerem Mert Senses, Katarzyna Ulewicz, Katarzyna Witt, Joanna Szymanska, Bartlomiej Taciak, Robert Klopfleisch, Eva Hellmén, Izabella Dolka, Ali O. Gure, Joanna Mucha, Mariusz Mikow, Slawomir Gizinski, Magdalena Krol

**Affiliations:** 1 Department of Physiological Sciences, Faculty of Veterinary Medicine, Warsaw University of Life Sciences, Nowoursynowska 159, 02-776 Warsaw, Poland; 2 Department of Molecular Biology and Genetics, Faculty of Science, SB Building, Bilkent University, 06800 Ankara, Turkey; 3 Institute of Veterinary Pathology, Freie Universitaet Berlin, Robert-von-Ostertag-Strasse 15, Building 31, 14163 Berlin, Germany; 4 Department of Anatomy, Physiology and Biochemistry, Swedish University of Agricultural Sciences, Box 7011, 75007 Uppsala, Sweden; 5 Department of Pathology and Veterinary Diagnostics, Faculty of Veterinary Medicine, Warsaw University of Life Sciences, Nowoursynowska 159, 02-776 Warsaw, Poland; 6 Veterinary Clinic ‘Elwet’, Niepodleglosci 24/30, 02-653 Warsaw, Poland; 7 Department of Large Animal Diseases with Clinic, Faculty of Veterinary Medicine, Warsaw University of Life Sciences, Nowoursynowska 100, 02-797 Warsaw, Poland

**Keywords:** microRNA, Canine mammary cancer, Human breast cancer

## Abstract

**Background:**

MicroRNAs may act as oncogenes or tumour suppressor genes, which make these small molecules potential diagnostic/prognostic factors and targets for anticancer therapies. Several common oncogenic microRNAs have been found for canine mammary cancer and human breast cancer. On account of this, large-scale profiling of microRNA expression in canine mammary cancer seems to be important for both dogs and humans.

**Methods:**

Expression profiles of 317 microRNAs in 146 canine mammary tumours of different histological type, malignancy grade and clinical history (presence/absence of metastases) and in 25 control samples were evaluated. The profiling was performed using microarrays. Significance Analysis of Microarrays test was applied in the analysis of microarray data (both unsupervised and supervised data analyses were performed). Validation of the obtained results was performed using real-time qPCR. Subsequently, predicted targets for the microRNAs were searched for in miRBase.

**Results:**

Results of the unsupervised analysis indicate that the primary factor separating the samples is the metastasis status. Predicted targets for microRNAs differentially expressed in the metastatic vs. non-metastatic group are mostly engaged in cell cycle regulation, cell differentiation and DNA-damage repair. On the other hand, the supervised analysis reveals clusters of differentially expressed microRNAs unique for the tumour type, malignancy grade and metastasis factor.

**Conclusions:**

The most significant difference in microRNA expression was observed between the metastatic and non-metastatic group, which suggests a more important role of microRNAs in the metastasis process than in the malignant transformation. Moreover, the differentially expressed microRNAs constitute potential metastasis markers. However, validation of cfa-miR-144, cfa-miR-32 and cfa-miR-374a levels in blood samples did not follow changes observed in the non-metastatic and metastatic tumours.

**Electronic supplementary material:**

The online version of this article (10.1186/s12885-017-3751-1) contains supplementary material, which is available to authorized users.

## Background

Mammary tumours occur spontaneously in dog and human populations [1]. Epidemiology of this disease is similar in both species, partly due to the dog being a companion animal, i.e. living in similar environmental conditions to humans. Canine and human mammary tumours are hormone-dependent and usually originate from epithelial tissue [2]. The most common histological type of malignant mammary tumours in dogs is complex carcinoma [3] and that of human breast cancer is invasive ductal carcinoma [4]. Canine mammary carcinoma frequently invades lymph nodes and metastasises to the lungs [5, 6], but rarely to the bones [6, 7]. Human breast cancer often spread to lymph nodes, lungs, bones and to the liver [8]. Many similar oncogenes were found for human breast cancer and canine mammary carcinoma, for instance oncogenic microRNAs [9]. Moreover, many changes in pathways related to mammary cancer (including KRAS, PTEN, PI3K/AKT, WNT-beta catenin and MAPK cascade) are common for both species [10]. All these molecular similarities made canine mammary cancer a good genetic model for human breast cancer [11].

Some microRNAs are up-regulated and some are down-regulated in cancer, which suggests that microRNAs may act as oncogenes or tumour suppressor genes [12]. Many microRNAs are located in fragile sites (FRAs) − preferential sites of alterations (e.g. amplification or deletion) in a genome. Hence, amplification of chromosomal regions containing oncogenic microRNAs and/or deletion of sites including suppressor microRNAs may lead to cancer development [13]. When showing the connection between microRNA expression and cancer it is also very important to establish microRNA’s functional role. For example, p53 activates the expression of miR-34a, which then promotes apoptosis [14]. MiR-27a inhibits the expression of the Sp repressor ZBTB10/RINZF [15], leading to the overexpression of Sp factors and, as a consequence, to the increase of Sp-dependent antiapoptotic and angiogenic molecules’ number, e.g. survivin and vascular endothelial growth factor (VEGF), responsible for cancer development [16]. MiR-10b suppresses the homeobox D10 (HOXD10). Expression of HOXD10 releases the pro-metastatic gene RHOC and results in tumour invasion and metastasis [17]. In general, all these findings suggest that microRNAs may serve as diagnostic and prognostic factors.

Expression profiles of a few microRNAs have been investigated in canine mammary cancer. Von Deetzen et al. compared the expression profiles of 16 microRNAs (miR-136, miR-143, let-7f, miR-29b, miR-145, miR-9, miR-10b, miR-203, miR-125b, miR-15a, miR-16, miR-21, miR-101, miR-210, miR-194 and miR-125a) in three types of canine mammary tumours (adenoma, non-metastasising carcinoma, metastasising carcinoma), lymph node metastases and in a normal mammary gland. One of their results was the higher expression level of miR-210 in all neoplastic tissues in comparison to the normal gland. They also found that miR-29b, miR-101, miR-143, miR-145 and miR-125a are down-regulated in metastatic sites when compared to the primary tumours. Further, they did not find any significant difference in miR-9, miR-10b, miR-15a, miR-16, miR-125b, miR-136 and let-7f expression levels among the examined groups [18].

Our study is the first to identify the expression profiles of 317 microRNAs in canine mammary tumours of different histological type, malignancy grade and clinical history (presence or absence of metastases) and in a control group (normal mammary gland samples). This work was performed using microarrays – a novel large-scale profiling method.

## Methods

### Tumour sample collection

Tumour samples were collected during mastectomy performed according to standard veterinary procedures. One half of every tumour was immersed in 10% neutral buffered formalin and stored at room temperature. The other was immersed in RNA*later*® Stabilization Solution (Ambion, USA) and stored at −80°C. The total number of obtained samples amounts to 171 (39 samples were received from veterinary clinics in Warsaw (Poland), 104 samples – from the Freie Universitaet Berlin (Berlin, Germany) and 28 samples – from the Swedish University of Agricultural Sciences (Uppsala, Sweden)). The samples from Germany and Sweden were shipped to Poland in RNA*later*® Stabilization Solution on dry ice. Radiography was used for the diagnosis of metastases for the cases in Poland. The samples from Sweden were obtained from dogs that died or were euthanized due to their mammary carcinoma. This was confirmed by a post-mortem examination or x-ray of the lungs and the latter was based on the information from the clinician or from the owner [19].

### Tumour classification and immunohistochemistry

Histological classification of the tumours was performed according to the World Health Organization (WHO) Histological Classification of Mammary Tumors in the Dog and Cat [20]. Grades of malignancy were allocated in accordance with the Nottingham method for human breast tumours, which is based on the assessment of three morphological features: mitotic counts, nuclear pleomorphism and tubule formation [21]. Tumoural characteristics of the samples No. 26–144 were assessed by immunohistochemical examination of cytokeratin, vimentin, smooth muscle actin, s100 protein and p63 protein expression.

For immunohistochemical analysis, tumour samples were embedded in paraffin. 3-μm-thick sections of the tumours were cut, fixed on slides and dried overnight at 37°C. After drying, slides were dewaxed in xylene, rehydrated in ethanol, boiled in 0.02 M citrate buffer (pH 6.0), washed in H_2_O_2_, washed with distilled water, washed in phosphate buffered saline (PBS) and incubated in 1–2% bovine serum albumin. Afterwards, sections were incubated overnight at 4°C in primary antibodies diluted in 1–2% bovine serum albumin. The following primary antibodies were used: Monoclonal Mouse Anti-Human Cytokeratin, Clone MNF116, 1:50 (Dako, Agilent Technologies, USA); Monoclonal Mouse Anti-Vimentin, Clone Vim 3B4, 1:100 (Dako); Monoclonal Mouse Anti-Human Actin (Muscle), Clone HHF35, 1:50 (Dako); Polyclonal Rabbit Anti-S100, 1:400 (Dako) and Monoclonal Mouse Anti-Human p63 Protein, 1:50 (Dako). After incubation in primary antibodies, slides were washed in PBS. Subsequently, staining was performed using EnVision™+ System-HRP (DAB) Kit (Dako). Tumour sections were incubated in Labelled Polymer-HRP (polymer conjugated with horseradish peroxidase enzyme) and in 3,3`-diaminobenzidine (DAB) chromogen (diluted according to the manufacturer’s protocol). After chromogen reaction, slides were washed under cold running water, stained with haematoxylin and eosin, washed again under cold running water and dehydrated in alcohol and in xylene. Coverslips on slides were fixed using Mounting Medium (Dako). Slides with coverslips were dried overnight at 37°C. Antigen spots were counted by a computer-assisted image analyser (Olympus Microimage™ Image Analysis, software version 4.0 for Windows, Japan).

### RNA isolation from tumour samples

RNA was isolated from tumour pieces with a diameter of 1 cm. Each piece was washed with RNase Away Reagent (Ambion) and disrupted in Tissue Lyser LT (QIAGEN, Germany) at 50 Hz for 30 min. After disruption, total RNA was isolated from samples using miRNeasy Mini Kit (QIAGEN) according to the manufacturer’s protocol. Isolated RNA was stored at −80°C. RNA quantity and contamination with proteins and organic compounds were examined using NanoDrop 2000 (NanoDrop, USA). RNA integrity was assessed using Agilent 2100 Bioanalyzer (Agilent Technologies, USA).

### MicroRNA microarray profiling

Samples (750 ng of total RNA) were labelled with fluorescent labels (examined samples with Hy3™ label − green fluorescence, reference samples with Hy5™ label − red fluorescence) using miRCURY LNA™ microRNA Hi-Power Labeling Kit, Hy3™/Hy5™ (Exiqon, Denmark) according to the manufacturer’s protocol. The Hy3™-labelled examined samples and Hy5™-labelled reference samples were mixed pair-wise and hybridized on miRCURY LNA™ microRNA Array 7th generation − hsa, mmu & rno (Exiqon) using Tecan HS4800TM Hybridization Station (Tecan, Austria). After hybridization, microarray slides were scanned and stored in an ozone free environment (ozone level below 2.0 ppb). Scanning was carried out using Agilent G2565BA Microarray Scanner (Agilent Technologies). Image analysis was performed using ImaGene 9.0 software (BioDiscovery, USA). Quantified signals were normalized using quantile normalization method.

Both unsupervised and supervised analyses of data were performed. Unsupervised analysis was carried out without dividing samples into groups and it includes Principal Component Analysis (PCA) and unsupervised hierarchical clustering (two-way hierarchical clustering). For supervised analysis, samples were divided into groups according to three factors: tumour type, malignancy grade, and metastasis. Unsupervised and supervised analyses of data were performed using BRB-ArrayTools, Version 4.3.2 (developed by Dr. Richard Simon and BRB-Array Tools Development Team). For the unsupervised analysis, the variances of microRNAs were calculated using Excel’s VAR.S function. For the supervised analysis, Significance Analysis of Microarrays (SAM) test, which sets estimate of False Discovery Rate for multiple testing, was applied. Results of the analyses are shown on heat maps and 3D–PCA plots. Heat maps were drawn using BRB-ArrayTools, 3D–plots − using Python programming version 3.4 [22] with the Matplotlib version 1.4.3 data visualization package [23]. To make heat maps, the normalized expression values of microRNAs were standardized by the Excel’s STANDARDIZE function, then on the heat map an expression level below the mean was represented by green colour and an expression level above the mean was represented by red colour.

### Validation of microarray results

For validation of microarray results were selected microRNAs showing more than 2-fold up- or down-regulation (LogFoldChange above +1.0 or below −1.0), statistically significant regulation (adjusted *p*-values <0.05) and average array signal intensity of the probes well above the background (in the range 7.5–14.5). The real-time quantitative PCR (RT-qPCR) method was applied for the validation. cDNA synthesis was carried out using Universal cDNA Synthesis Kit (Exiqon) according to the manufacturer’s protocol in Eppendorf MasterCycler Personal thermal cycler (Eppendorf, Germany). RT-qPCR was performed using LNA™ PCR primer sets (Exiqon) and ExiLENT SYBR® Green master mix (Exiqon) according to the manufacturer’s protocol in Stratagene Mx3005P qPCR System (Agilent Technologies). Target sequences for the primer sets are shown in Additional file 1. The results of RT-qPCR were analysed using GenEx 6 software (Exiqon).

### Validation of selected targets for microRNAs deregulated in metastatic canine mammary cancer

The following 17 genes were selected for validation of predicted targets for microRNAs deregulated in metastatic canine mammary cancer: CDC6, CCNE1, MYBL2, PDCD10, ERBB2IP, SON, STK4, CDC27, PRC1, CDC37, TTK, SKIL, BUB3, SPIN1, EEF2, ACTB and HPRT. The RT-qPCR method was applied for the validation. cDNA synthesis was performed using High Capacity RNA-to-cDNA Kit (Life Technologies, USA) according to the manufacturer’s protocol in MasterCycler® pro PCR System (Eppendorf). RT-qPCR was carried out using Oligo.pl primer sets (Oligo, Poland) and SYBR® Select Master Mix (Life Technologies) according to the manufacturer’s protocol in Stratagene Mx3005P qPCR System (Agilent Technologies). The primers’ sequences are shown in Additional file 2. The results of RT-qPCR were analysed using GraphPad Prism 6 (GraphPad Software, USA).

### Blood samples

Whole blood samples were obtained from 50 female dogs diagnosed with canine mammary tumours. All these samples were collected during cephalic vein catheterization prior to mastectomy in the Department of Small Animal Diseases with Clinic (Faculty of Veterinary Medicine, Warsaw University of Life Sciences) and in two private veterinary clinics in Warsaw except a bitch that was not qualified for surgery due to lung metastasis. From this patient, blood was taken from the cephalic vein by catheterization before euthanasia. In brief, 38 dogs with non-metastatic tumours (10 benign and 28 malignant tumours in various stages) and 12 dogs with tumour recurrence or metastasis were qualified for this study. Detailed characteristics of all the samples are included in Additional file 3.

For a control group, 12 blood samples were collected from healthy bitches during routine veterinary examination before ovariohysterectomy in two private veterinary clinics in Warsaw. Patients with possible diseases and pathological stages, which might influence the study and its results, were excluded.

All dogs underwent standard clinical examination before the procedure, including: the patient’s history, complete physical examination, documentation of tumour characteristics, haematological examination, serum biochemistry profile and three thoracic radiographic projections – right, left lateral and dorsoventral. Four millilitres of blood were collected into 6 ml K_2_EDTA plastic tubes (BD Vacutainer) and centrifuged on the same day at 4000 RPM for 15 min at 4 °C. Plasma was next carefully aspirated and transferred into a new tube and centrifuged again under the same conditions. Finally, the supernatant was transferred into a new tube and stored at −80 °C until RNA isolation.

### RNA isolation from plasma samples

MicroRNA was extracted using QIAamp Circulating Acid Kit (QIAGEN) according to the manufacturer’s protocol for 1 ml of plasma. In the last step of the procedure, microRNA was suspended in 40 μl of elution buffer AVE. The quantity of microRNA was measured using NanoDrop Spectrophotometer (NanoDrop Technologies, USA) whereas RNA quality and integrity were assessed using BioAnalyzer (Agilent, USA). Only samples with a RIN > 8 were taken to the further study.

### cDNA synthesis and quantitative real time PCR for plasma microRNAs

Four commercially available microRNA LNA PCR primer sets (cfa-miR-144, cfa-miR-32, cfa-miR-374a and hsa-miR-1246 (Exiqon)) were selected as metastasis-specific and used to evaluate microRNA levels in each plasma sample. Additionally, four microRNAs were chosen as controls (according to Blondal et al.) for all the samples to investigate possible haemolysis and erythrocyte contamination, which might alter microRNA levels in samples [24]. Two microRNAs affected (hsa-miR-425-5p and hsa-miR-486-3p) and two non-affected by haemolysis (hsa-miR-744-5p and hsa-miR-340-5p) were selected [25]. However, in the final calculations, the most sensitive and detectable microRNAs in all the samples were used (i.e. hsa-miR-486-3p and hsa-miR744). Target sequences for the primer sets are shown in Additional file 4.

The formula proposed by Blondal et al. identifies haemolysis based on the value obtained by substracting dCT hsa-miR-486-3p from dCT hsa-miR-744. Samples with a ddCT >5 are considered as haemolysed and samples with a ddCT between 7 and 8 are considered as strongly haemolysed [24].

cDNA synthesis was performed using Universal cDNA Synthesis Kit (Exiqon) according to the manufacturer’s protocol in Eppendorf Master Cycler Personal thermal cycler (Eppendorf, Germany). Samples were further frozen and stored at −20 °C. The synthetized cDNA was diluted 1:40 and used within 24 h for qRT-PCR carried out using ExiLENT SYBR® Green master mix (Exiqon). 10 μl of a reaction mixture consists of 5 μl of PCR Master Mix, 1 μl of PCR primer mix and 4 μl of diluted cDNA template. Each reaction was run in triplicate on a 96-well plate using Stratagene Mx3005P qPCR System (Agilent Technologies). Results of qRT-PCR were calculated using the comparative Ct method [26] and statistically analysed by Prism version 6.00 software (GraphPad Software, USA). An unpaired, non-parametric Mann-Whitney test was applied to compare the difference of microRNAs expression between the non-metastatic and metastatic group. Statistical significance was defined as *p*-value <0.05. Due to the magnitude and range of observed results, the data was log-transformed for analysis.

## Results

### Sample characteristics

A total of 171 samples were included in this study, 146 of which were canine mammary tumours (30 benign tumours and 116 malignant tumours) and 25 were normal mammary gland samples. The group of malignant tumours consisted of 115 carcinoma samples and one carcinosarcoma case. The malignant tumours were of different histological subtypes (histological classification of the samples is included in Table 1), grades of malignancy (grade I − 27 samples, grade II − 29 samples, grade III − 27 samples, unknown − 33 samples) and clinical histories (presence of metastases − 58 samples, absence of metastases − 49 samples, unknown − 9 samples). Detailed characteristics of all the samples included in the study are shown in Additional file 5.

**Table 1:** Histological classification of the tumour samples (summary)

Control/Tumour type	Histological type	No.	Σ
Control		25	25
Benign tumour	Simple adenoma	3	30
	Complex adenoma	16	
	Atypical adenoma	1	
	Atypical simple adenoma	1	
	Atypical complex adenoma	1	
	Basaloid adenoma	1	
	Atypical papilloma	3	
	Benign mesenchymal tumour	2	
	Benign mixed tumour	1	
	Simple adenoma + Complex adenoma	1	
Malignant tumour	Solid carcinoma	22	113
	Simple carcinoma	1	
	Complex carcinoma	16	
	Tubulopapillary carcinoma	30	
	Noninfiltrating carcinoma	3	
	Comedocarcinoma	16	
	Squamous cell carcinoma	3	
	Basaloid carcinoma	1	
	Anaplastic carcinoma	3	
	Lipid-rich carcinoma	2	
	Mucinous carcinoma	1	
	Scirrhous carcinoma	3	
	Carcinoma	1	
	Special type of carcinoma	1	
	Carcinosarcoma	1	
	Tubulopapillary carcinoma/Noninfiltrating carcinoma	1	
	Solid carcinoma / Lipid-rich carcinoma	1	
	Tubulopapillary carcinoma + Solid carcinoma	2	
	Mucinous carcinoma + Tubulopapillary carcinoma	1	
	Tubulopapillary carcinoma + Bi-phasic carcinoma	1	
	Unknown	3	
Malignant tumour + benign tumour	Tubulopapillary carcinoma + Complex adenoma	1	2
	Tubulopapillary carcinoma + Noninfiltrating carcinoma + Benign mixed tumour	1	
Malignant tumour + hyperplasia	Complex carcinoma + Mammary ductal ectasia	1	1

### MicroRNA microarray analysis

The technical data quality assessment showed that the sample labelling with Hy3™ and Hy5™ fluorescent labels was successful as all capture probes for the control spike-in oligonucleotides produced signals in the expected range. Subsequently, a total of 317 microRNAs were used for threshold filtering and 122 probes were discarded by the filtering procedure. The obtained number of present calls for the samples was within the expected range.

**Fig. 1:**
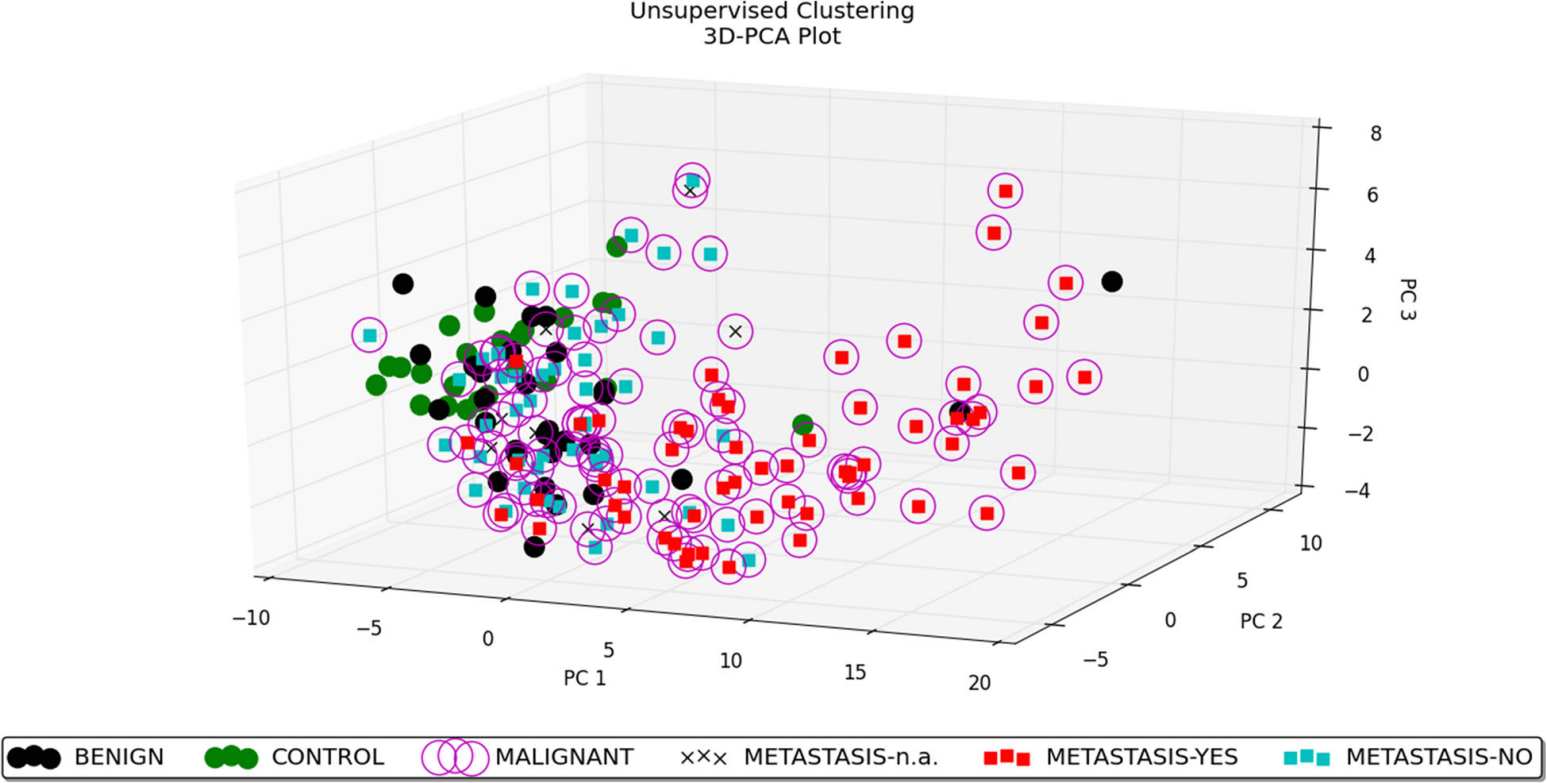
3D–PCA plot − unsupervised analysis. PCA performed on all the samples and on the top 50 microRNAs with the highest standard deviation. The normalized log-transformed Hy3 values were used for the analysis. The features were shifted to be zero centred, (i.e. the mean value across samples was shifted to 0) and scaled to have unit variance (i.e. the variance across samples was scaled to 1) before the analysis. PCA plot reveals the distinct sample clusters for metastatic tumours, non-metastatic tumours and the control group

**Fig. 2:**
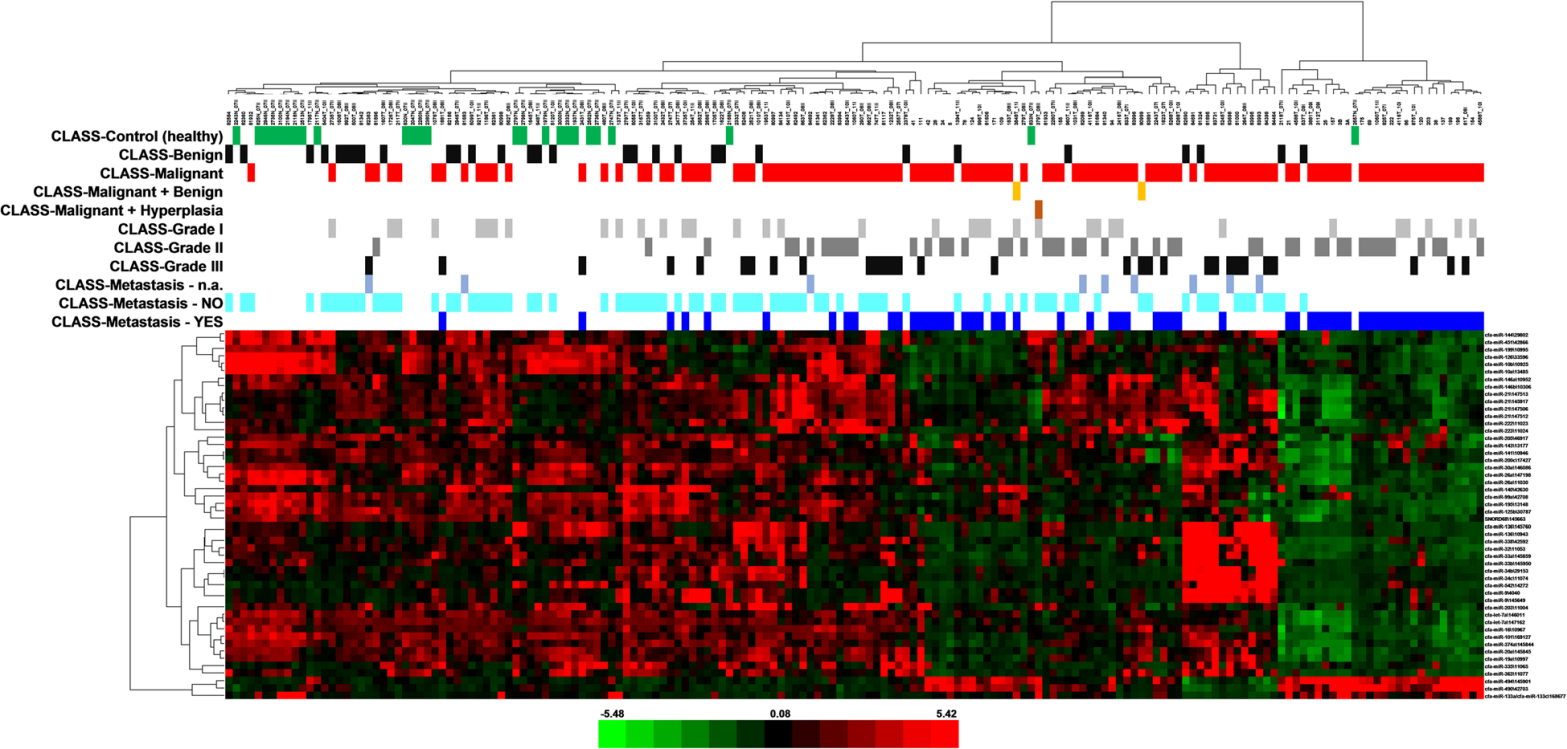
Heat map − unsupervised hierarchical clustering. Clustering performed on all the samples and on the top 50 microRNAs with the highest standard deviation. The normalized log-transformed Hy3 values were used for the analysis. The colour scale illustrates the relative expression level of a microRNA across all samples: green colour represents an expression level below the mean and red colour represents an expression level above the mean. Legend: n.a. − data not available

Both unsupervised and supervised analyses of data were performed. Unsupervised analysis includes Principal Component Analysis and unsupervised hierarchical clustering (two-way hierarchical clustering). Principal Component Analysis was conducted to reduce the dimensions of large data sets and to explore the naturally arising sample classes based on the expression profile. By including the top 50 microRNAs with the largest variation across all the samples, we obtained an overview of how the samples clustered based on this variance. This led to the separation of the samples in different regions of a PCA plot corresponding to their biology. The result of PCA is presented as a 3D–PCA plot (Fig. 1). The 3D–PCA plot reveals the distinct sample clusters for metastatic tumours, non-metastatic tumours and the control group. Unsupervised hierarchical clustering (two-way hierarchical clustering of microRNAs and samples) was performed using the complete-linkage method together with the Euclidean distance measure. The result of this analysis is shown in Fig. 2. In general, the unsupervised analysis shows that the primary factor separating the samples is the metastatic status.

**Fig. 3:**
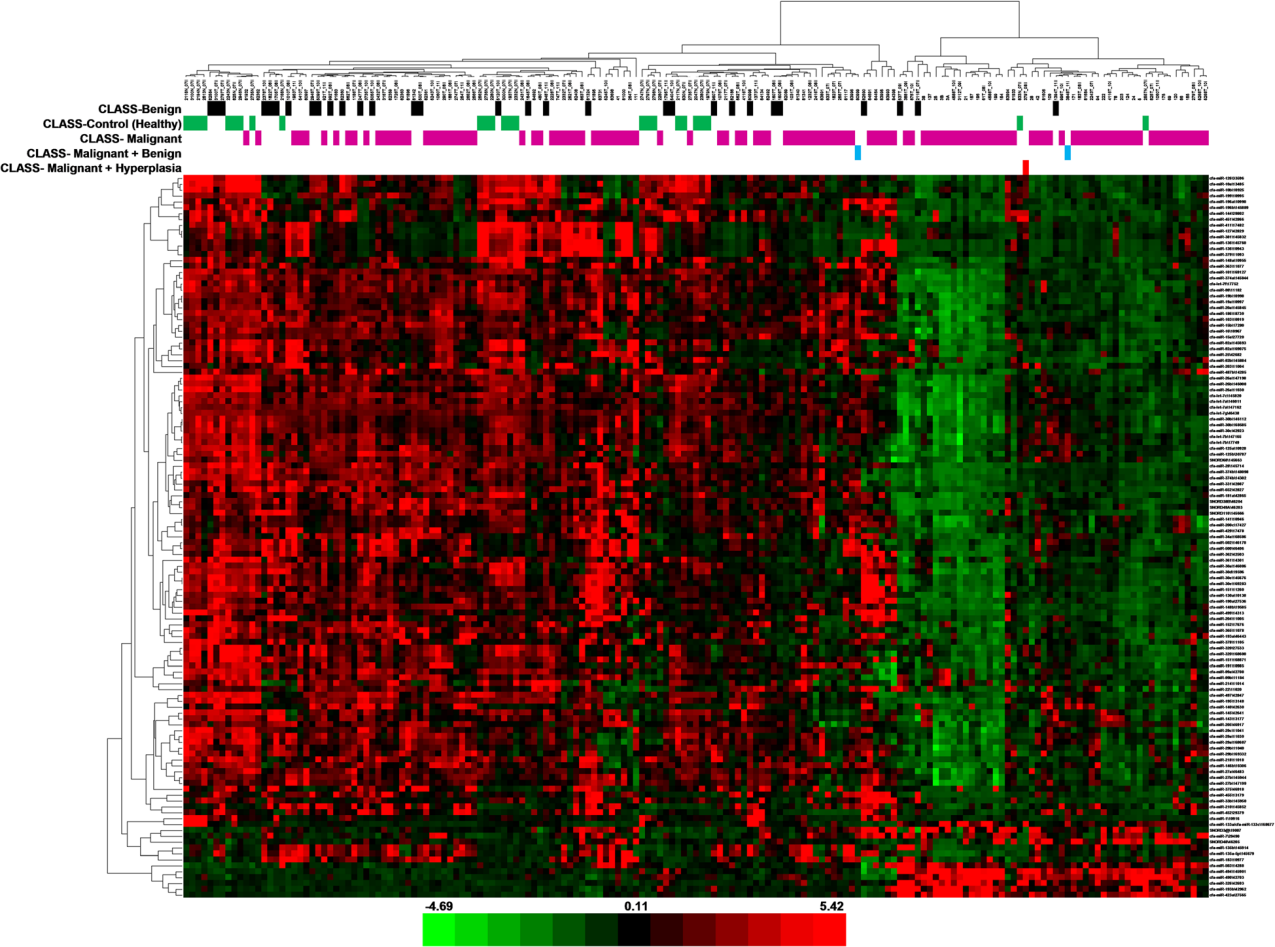
Heat map − tumour type. The scaled expression of the differentially expressed microRNAs for 171 samples and the relationship among the samples in terms of microRNAs found to be differentially expressed for the tumour type factor; Significance Analysis of Microarrays (SAM) test; the colour scale illustrates the relative expression level of a microRNA across all samples: green colour represents an expression level below the mean and red colour represents an expression level above the mean. The most distinct differences in microRNA profile are between the control and malignant group − microRNAs mostly down-regulated in the malignant group

**Fig. 4:**
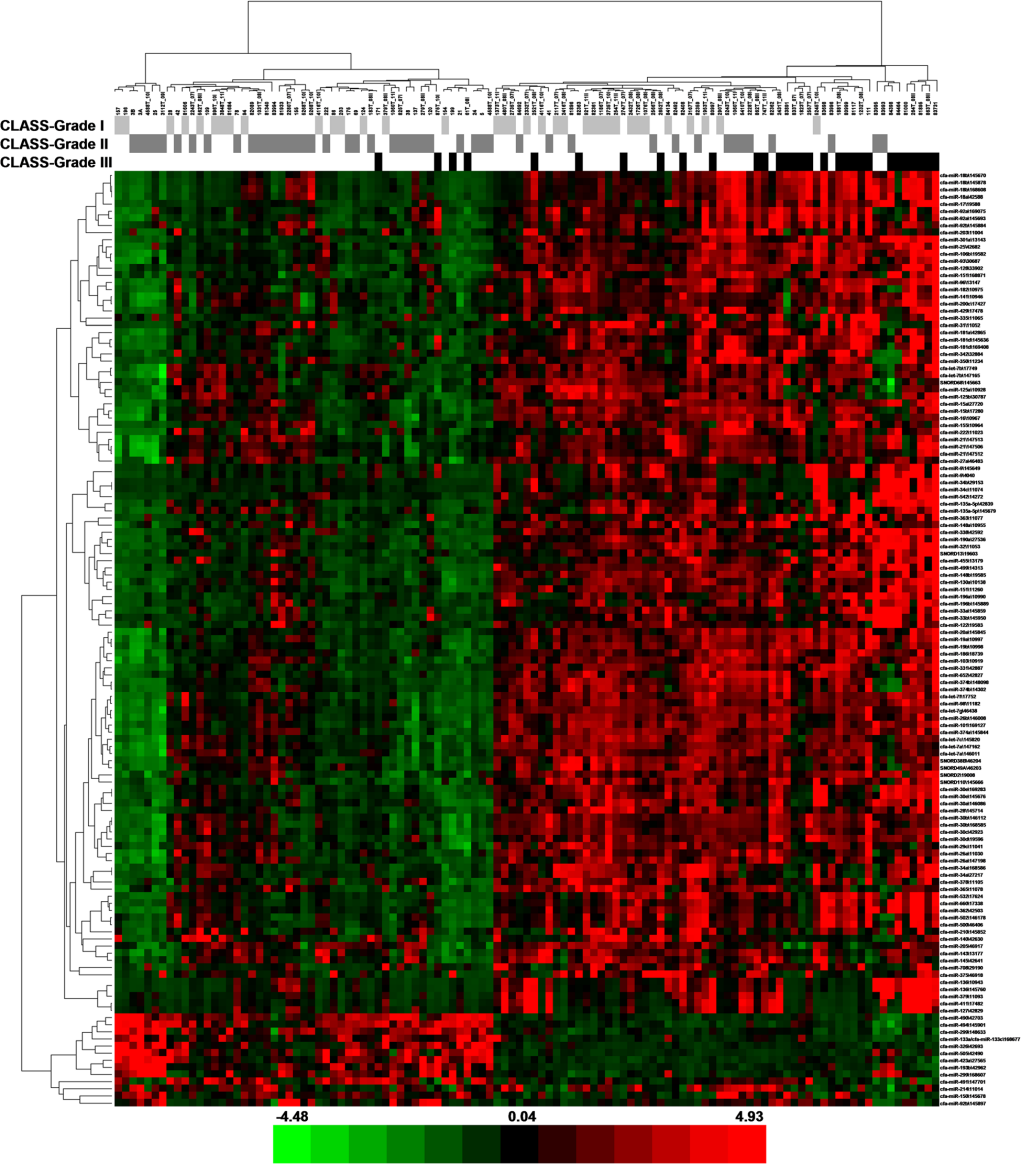
Heat map − grade of malignancy. The scaled expression of the differentially expressed microRNAs for 111 samples and the relationship among the samples in terms of microRNAs found to be differentially expressed for the malignancy grade factor; Significance Analysis of Microarrays (SAM) test; the colour scale illustrates the relative expression level of a microRNA across all samples: green colour represents an expression level below the mean and red colour represents an expression level above the mean. MicroRNAs are mostly up-regulated in the grade III group in comparison with the grade II group

**Fig. 5:**
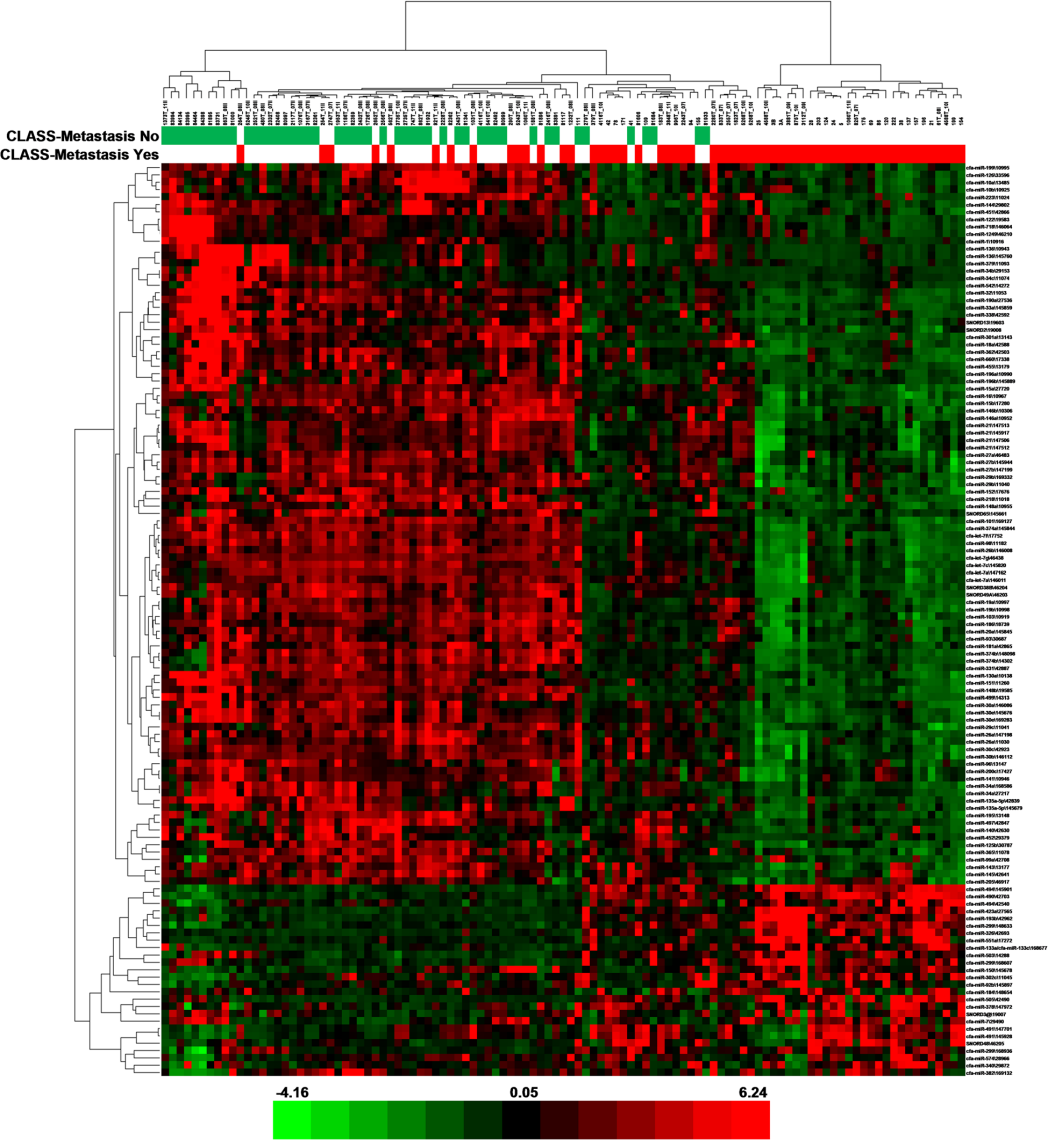
Heat map − metastasis. The scaled expression of the differentially expressed microRNAs for 107 samples and the relationship among the samples in terms of microRNAs found to be differentially expressed for the metastasis factor; Significance Analysis of Microarrays (SAM) test; the colour scale illustrates the relative expression level of a microRNA across all samples: green colour represents an expression level below the mean and red colour represents an expression level above the mean. MicroRNAs are mostly down-regulated in the metastatic group

**Fig. 6:**
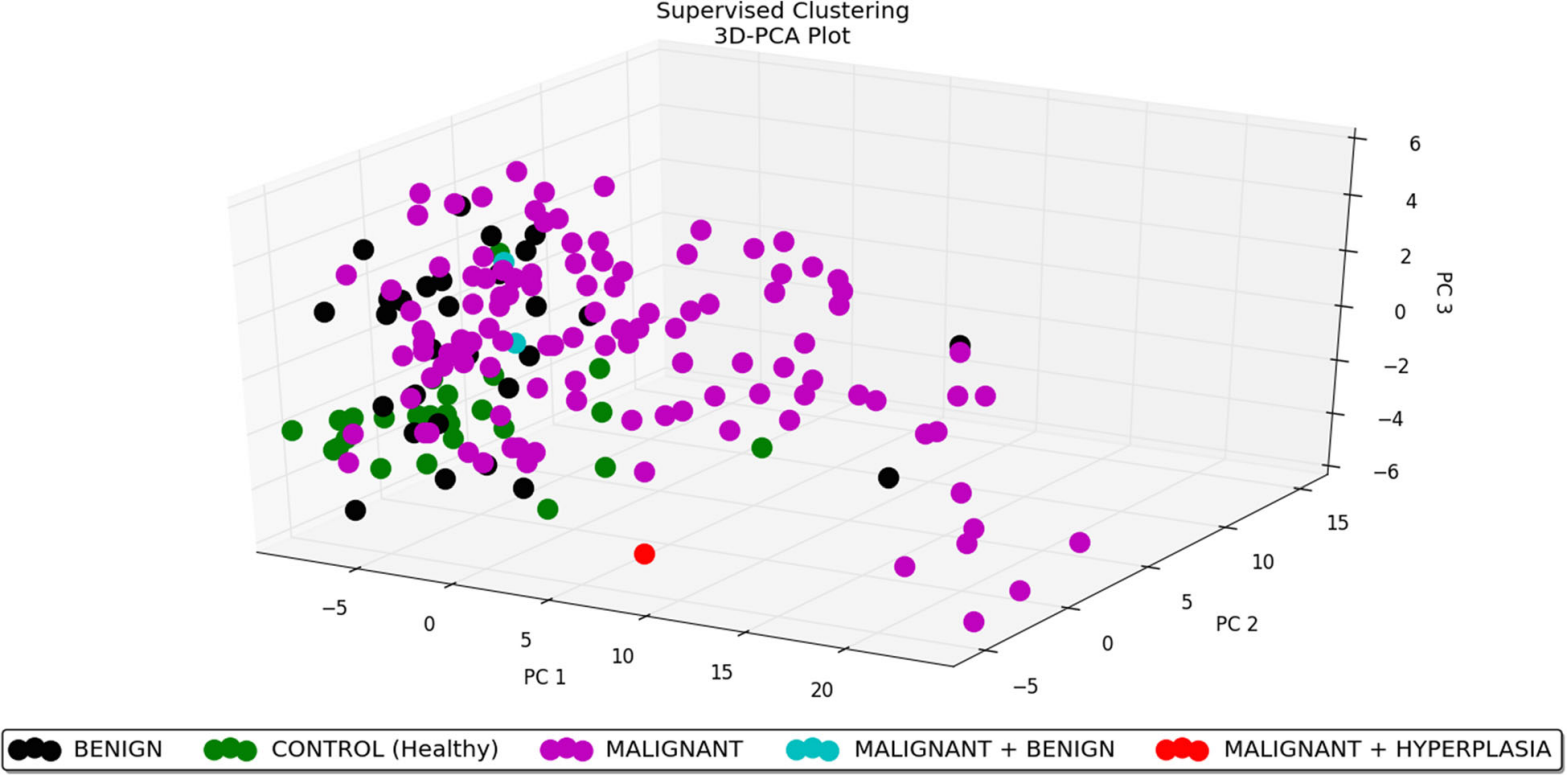
3D–PCA plot − tumour type. PCA plot performed on 171 samples and 123 differentially expressed microRNAs for the tumour type factor; Significance Analysis of Microarrays (SAM) test. PCA plot reveals the distinct sample clusters for malignant tumours, benign tumours and the control group

**Fig. 7:**
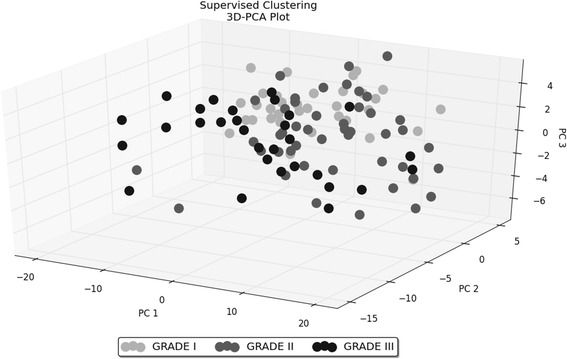
3D–PCA plot − grade of malignancy. PCA plot performed on 111 samples and 131 differentially expressed microRNAs for the malignancy grade factor; Significance Analysis of Microarrays (SAM) test

**Fig. 8:**
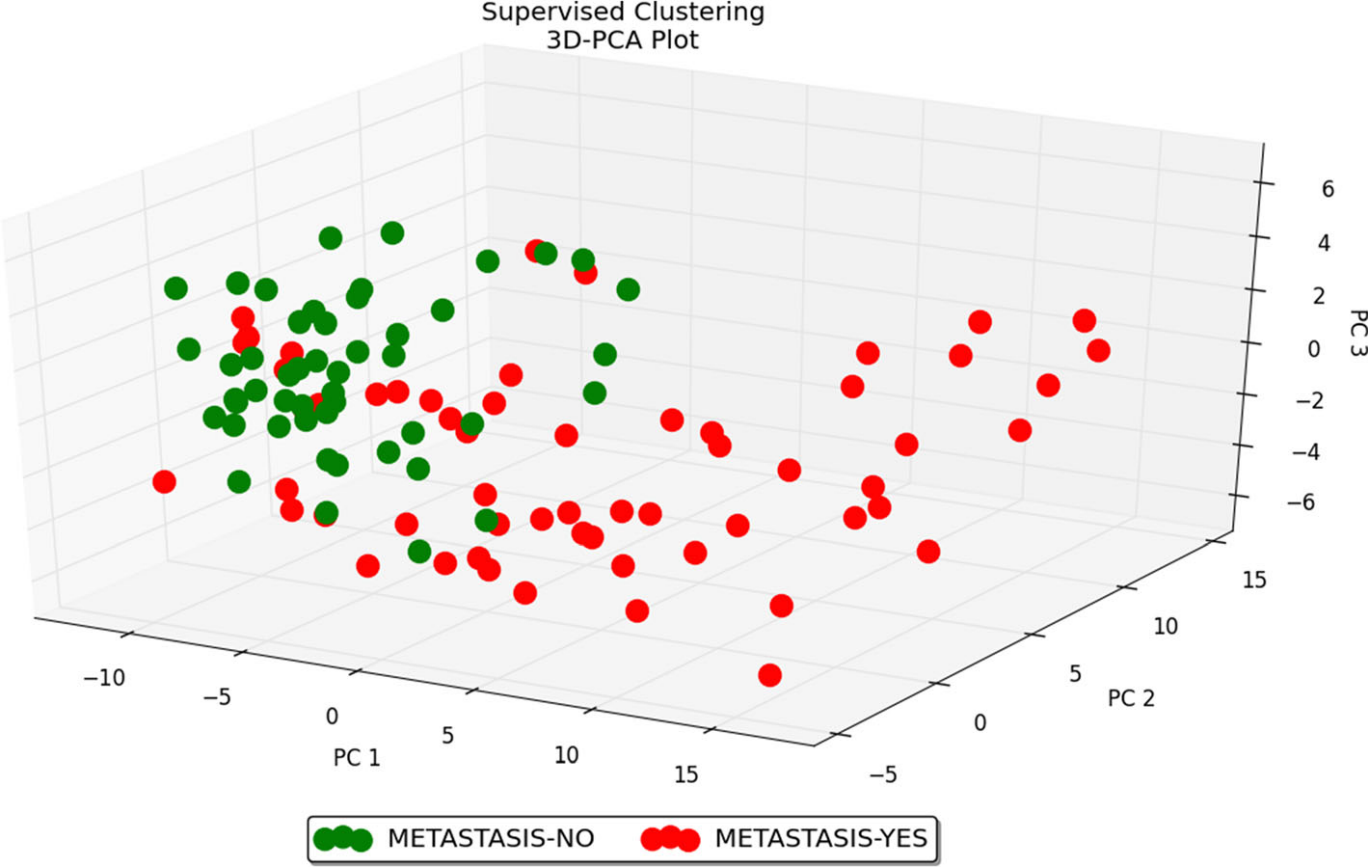
3D–PCA plot − metastasis. PCA plot performed on 107 samples and 124 differentially expressed microRNAs for the metastasis factor; Significance Analysis of Microarrays (SAM) test. PCA plot reveals the distinct sample clusters for metastatic tumours and non-metastatic tumours

For supervised analysis, samples were divided into groups according to three factors: tumour type, malignancy grade and metastatic status (the histological subtype was not used as a factor for any analysis because of a large number of groups with widely varying sizes). Significance Analysis of Microarrays test for each of the three factors was performed. For the tumour type factor, a subset of 123 microRNAs was identified out of the total of 195 analysed microRNAs that are significantly differentially expressed in different groups (the controls, benign tumours and malignant tumours). Of these 123 microRNAs, 84 miRNAs were down-regulated in the malignant tumour group in comparison with the control group, seven miRNAs were up-regulated in the malignant group compared to the controls, 20 miRNAs were down-regulated in the malignant tumours when compared to the benign tumour group, three miRNAs were up-regulated in the malignant group in comparison with the benign group, eight miRNAs were up-regulated in the benign tumours compared to the controls and one miRNAs was down-regulated in the benign tumour group when compared to the controls. The results of this analysis are shown in Additional file 6. One of the samples used in this analysis was marked as ‘malignant + hyperplasia’, as it was classified as a mixed histological type. The microRNA profile of this sample was mostly similar to the malignant group, because the specimen included probably the malignant part of a tumour as the main component. For the grade of malignancy, 131 differentially expressed microRNAs were identified (Additional file 7) in the following groups: grade I, grade II and grade III (including 13 miRNAs down-regulated in the grade II group when compared to the grade I group, 95 miRNAs up-regulated in the grade III group in comparison with the grade II group, one miRNAs down-regulated in the grade III group when compared to the grade I group, ten miRNAs down-regulated in the grade III group in comparison with the grade II group, ten miRNAs up-regulated in the grade III group when compared to the grade I group and two miRNAs up-regulated in the grade II group compared to the grade I group); and for the metastasis factor − 124 miRNAs (Additional file 8) in the metastatic and non-metastatic group (including 98 miRNAs down-regulated and 26 miRNAs up-regulated in the metastatic group in comparison with the non-metastatic group). In general, the most distinct differences in microRNA profiles are between the control and malignant group for the tumour type factor (microRNAs mostly down-regulated in the malignant group) and between the metastatic and non-metastatic group for the metastasis factor (microRNAs mostly down-regulated in the metastatic group). To enable quick visual identification of microRNAs displaying large-magnitude changes that are also statistically significant, the expression data were plotted in heat maps (tumour type − Fig. 3, grade of malignancy − Fig. 4, metastasis − Fig. 5). For these microRNAs, PCA plots were also performed (tumour type − Fig. 6, grade of malignancy − Fig. 7, metastasis − Fig. 8).

### Validation of microarray results

The following ten microRNAs were selected for validation of microarray results: cfa-let-7c, cfa-miR-10b, cfa-miR-26a, cfa-miR-26b, cfa-miR-29c, cfa-miR-30a, cfa-miR-30b, cfa-miR-30c, cfa-miR-148a and cfa-miR-299. Average array signal intensity of the probes, *p*-values and fold changes for these microRNAs can be found in Additional file 8. The validation was performed on 47 samples including five controls, five benign tumours, ten malignant non-metastatic tumours and 27 malignant metastatic tumours. The validation results are shown in Table 2. All the validated microRNAs are significantly differentially expressed among the examined groups. However, the distinction among the expression levels of these microRNAs in the groups is not as marked as in the microarray analysis.

**Table 2:** Validation of microarray results in tumour samples

microRNA	microarray *p*-value	RT-qPCR *p*-value	Average RT-qPCR repeats, normalized			
			Control	Benign	Malignant non-metastatic	Malignant metastatic
let-7c	< 1e-07	0.02892256	2.066592593	1.551592593	0.885092593	0
miR-26b	< 1e-07	0.00001507	1.56060648	0.04060648	−0.57989352	−1.02131944
miR-26a	< 1e-07	0.00003571	1.11365741	0.50621296	−0.58834259	−1.03152778
miR-30a	3.00E-07	0.00367713	−0.09936889	0.71563112	0.08413112	−0.70039334
miR-148a	3.00E-07	0.04282469	0.32479167	0.55945833	−0.50670833	−0.37754167
miR-29c	5.00E-07	0.00019157	0.79212037	0.30412037	−0.25387963	−0.84236111
miR-30c	2.70E-06	0.00133947	0.2615463	0.59621296	0.0315463	−0.88930556
miR-299	5.40E-06	0.04632324	1.899074074	0.425074074	2.399074074	0
miR-10b	6.00E-06	0.01635353	0.9961408	0.07058524	−0.46735921	−0.59936683
miR-30b	1.74E-05	0.00557426	0.61328913	0.43528913	−0.40171087	−0.6468674

one-way ANOVA p-values for microarray results and RT-qPCR results; RT-qPCRs performed for 10 microRNAs on 47 samples (5 controls, 5 benign tumours, 10 malignant non-metastatic tumours and 27 malignant metastatic tumours)

### Validation of selected targets for microRNAs deregulated in metastatic canine mammary cancer

**Fig. 9:**
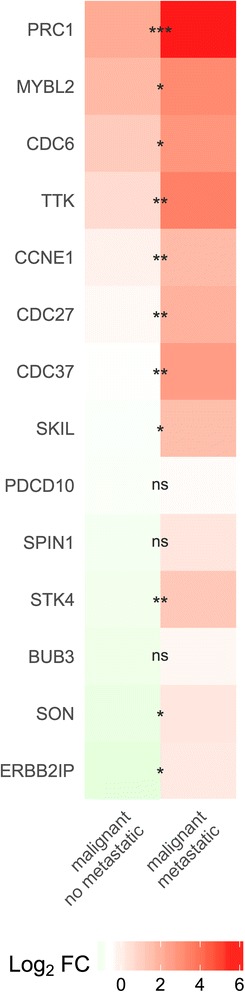
Heat map − selected targets for microRNAs deregulated in metastatic canine mammary cancer. The scaled expression of CDC6, CCNE1, MYBL2, PDCD10, ERBB2IP, SON, STK4, CDC27, PRC1, CDC37, TTK, SKIL, BUB3 and SPIN1. The analysis performed on ten malignant non-metastatic tumours and ten malignant metastatic tumours. Statistical analysis was made on Ct values normalized with a housekeeping gene; one-way ANOVA followed by Tukey’s HSD post hoc tests. Legend: log2 FC – log base 2 from fold change, * – *p* value <0.05, ** – *p* value <0.01, *** – *p* value <0.001

For validation of predicted targets for microRNAs deregulated in metastatic canine mammary cancer were selected 14 genes, which expression differ in the metastatic and non-metastatic group (CDC6, CCNE1, MYBL2, PDCD10, ERBB2IP, SON, STK4, CDC27, PRC1, CDC37, TTK, SKIL, BUB3 and SPIN1). Three housekeeping genes (EEF2, ACTB, and HPRT) were used as controls. The function of selected target genes and the list of microRNAs, which regulate their expression, are included in Additional file 9. The validation was performed on 20 samples (ten malignant non-metastatic tumours and ten malignant metastatic tumours). The validation results are shown in Fig. 9 and in Table 3. Klopfleisch et al. found the higher expression of CDC6, CCNE1, MYBL2, PDCD10, ERBB2IP, SON, STK4, CDC27, PRC1, CDC37, TTK, SKIL, BUB3 and SPIN1 in metastatic canine mammary cancer in comparison with non-metastatic canine mammary cancer [27]. Our results show the statistically significant up-regulation of CDC6, CCNE1, MYBL2, ERBB2IP, SON, STK4, CDC27, PRC1, CDC37, TTK and SKIL in the metastatic group when compared to the non-metastatic group.

**Table 3:** Validation of selected targets for microRNAs deregulated in metastatic canine mammary cancer

Gene	Mean difference	Lower	Upper	*p*-value
CDC6	1.662166667	0.296219571	3.028113763	0.014739925
CCNE1	1.805333334	0.698148282	2.912518385	0.00111525
MYBL2	1.469333333	0.095498643	2.843168024	0.034267
PDCD10	0.315	−1.466393723	2.096393723	0.899858502
ERBB2IP	1.863333333	0.181873946	3.544792721	0.027609459
SON	1.691333333	0.149694553	3.232972114	0.029382504
STK4	2.356333333	0.708656745	4.004009921	0.004014445
CDC27	2.316333334	0.684213855	3.948452812	0.004297527
PRC1	3.368333333	1.644772232	5.091894434	0.000132898
CDC37	3.250833333	1.273918023	5.227748644	0.001019454
TTK	2.8475	0.856445769	4.838554231	0.004013127
SKIL	2.291666667	0.327926258	4.255407076	0.019726893
BUB3	0.976666667	−0.467839044	2.421172377	0.232448632
SPIN1	1.248333333	−0.448209573	2.944876239	0.180817071

The analysis performed on ten malignant non-metastatic tumours and ten malignant metastatic tumours. Statistical analysis was made on Ct values normalized with a housekeeping gene; one-way ANOVA followed by Tukey’s HSD post hoc tests

### Validation of selected microRNAs levels in plasma samples as cancer markers

Three of the most down-regulated miRNAs in the metastatic group, revealed in tumour samples in our microarray analysis (cfa-miR-144, cfa-miR-32 and cfa-miR-374a), and hsa-miR-1246, known for its deregulation in plasma from human breast cancer patients [28], were chosen for the evaluation in plasma samples. Thirty-five out of fifty examined plasma samples were derived from the same dogs which tumours were used for the microarray analysis.

**Fig. 10:**
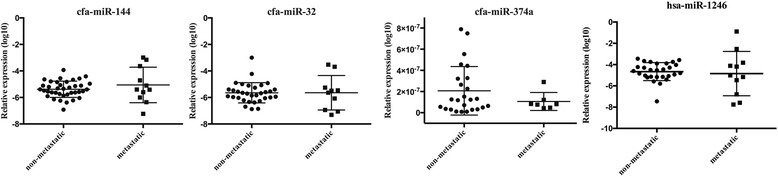
Expression of selected microRNAs in plasma samples from dogs with non-metastatic and metastatic tumours. Relative expression of cfa-miR-144, cfa-miR-32 cfa-miR-374a and hsa-miR-1246 in plasma samples from dogs with non-metastatic and metastatic tumours. The statistical analysis was performed using Prism version 5.00 software (GraphPad Software, USA). An unpaired, non-parametric Mann-Whitney test was applied. Values are mean ± SD

RT-PCR results for these four microRNAs in plasma demonstrated no significant differences in expression level between the metastatic and non-metastatic group. Moreover, plasma levels of these microRNAs did not differ significantly when compared to those in healthy dogs. *P*-values vary from 0.6 in cfa-miR-144, 0.89 in cfa-miR-32, to 0.27 in cfa-miR-374a in contrast to <1e^−07^ in tumour samples and fold change 4.74, 3.54 and 3.24, respectively. P-value for hsa-miR-1246 amounts to 0.67. The results are shown in Fig. 10.

### Evaluation of haemolysis risk in plasma samples

Results of hsa-miR-744-5p and hsa-miR-486-3p expression subtraction revealed that five out of 62 plasma samples show a minor risk of erythrocyte contamination (ranging from 5.01 to 5.7). However, these samples were not excluded from the analysis due to the lack of variation in the levels of investigated microRNAs when compared to the other samples and also occurrence of microRNAs, which are not characteristic for red blood cells.

## Discussion

The results of this study are largely in line with the findings of von Deetzen’s group [18]. We also found the significant down-regulation of miR-29b, miR-101, miR-143, miR-145 and miR-125a in a metastatic group in comparison with benign tumours. Higher expression of miR-210 in neoplasms than in a control group and up-regulation of miR-21 in non-metastasising tumours when compared to normal mammary tissue were similar in the two studies as well. However, there are some discrepancies between our findings and those of von Deetzen’s group. For instance, our results show that the expression of miR-203 is down-regulated in benign tumours in comparison with a control group. Von Deetzen et al. found that the expression level of this microRNA is higher in adenoma when compared to normal mammary tissue. Our findings revealed a gradual decrease in miR-10b, miR-125b, miR-136 and let-7f expression levels from normal mammary tissue, through benign tumours and non-metastatic malignant tumours, to metastatic tumours. Von Deetzen’s group observed no significant difference in the expression profiles of these microRNA among the examined tissues [18]. The discrepancies between the two studies may be caused by the fact that one microRNA can have many target genes and some tumours with the same histopathological diagnosis can be the result of different derangements of cellular pathways. Among the microRNAs for which the findings of the two groups differ, we found target genes in canine mammary cancer for miR-10b, miR-125b and let-7f (the targets together with their function are included in Additional file 9). The target genes for these microRNAs are engaged in cell cycle regulation, cell differentiation and receptors function. Up-regulation of these genes leads to deregulation of cellular processes and may be a reason for cancer development. However, the discrepancies between the results of this work and the findings of von Deetzen’s group may be caused by the differences in the sample types and in the data analysis used in these two studies. Von Deetzen et al. performed their experiments on three types of canine mammary tumours (adenoma, non-metastasising carcinoma and metastasising carcinoma) and analysed all these types separately. We used many histological types of benign, malignant non-metastatic and malignant metastatic tumours and combined together the histological types in the analysis of data.

### Predicted targets for microRNAs deregulated in metastatic canine mammary cancer

Klopfleisch et al. compared the global gene expression of metastatic and non-metastatic canine mammary carcinomas. They found 1011 differentially expressed genes, 744 of which were up-regulated and 267 were down-regulated. Among up-regulated genes were those engaged in cell cycle regulation, protein folding, proteasomal degradation and matrix modulation whereas among down-regulated ones where those which play roles in differentiation, growth factor pathways and actin organization [27]. We searched in miRBase [29] for predicted targets of microRNAs that we found differentially expressed in metastatic when compared to non-metastatic canine mammary cancer. Subsequently, we checked if these targets were among the genes deregulated in metastatic mammary cancer (described by Klopfleisch et al. [27]) and whether the expression profiles correlated i.e. under-expression of a microRNA suggests over-expression of its target gene and vice versa. Consequently, we found 44 microRNAs with predicted targets in metastatic canine mammary cancer. Forty-three of these microRNAs are down-regulated whereas one is up-regulated. A list of these 44 microRNAs together with their targets’ names and function is included in Additional file 9. Among the targets are mostly genes engaged in cell cycle regulation, cell differentiation and DNA-damage repair, which are all key processes in tumorigenesis.

### Common microRNAs deregulated in canine mammary cancer and human breast cancer

Seventeen out of all the microRNAs, which we found deregulated in canine mammary cancer, were also previously described as deregulated in human breast cancer. Twelve of them (miR-10b, miR-15a, miR-19a, miR-26b, miR-30a, miR-30c, miR-125a, miR-125b, miR-148a, miR-148b, miR-195 and miR-320) are down-regulated both in dogs and in humans whereas one (miR-494) is up-regulated in both species and four (miR-29a, miR-181a, miR-196a and miR-374a) are down-regulated in dogs but up-regulated in humans. Target genes of these 17 microRNAs are mostly engaged in cell cycle regulation, apoptosis and angiogenesis [30–45]. A full list of common microRNAs deregulated in canine mammary cancer and human breast cancer together with the target genes and their function is shown in Table 4.

**Table 4:** Common microRNAs deregulated in canine mammary cancer and human breast cancer [30–45]

microRNA	Canine mammary cancer	Human breast cancer	Target genes	Targets’ function
miR-10b	down-regulated	down-regulated	BUB1, PLK1, CCNA2	cell cycle regulation
miR-15a	down-regulated	down-regulated	CCNE1	cell cycle regulation
miR-19a	down-regulated	down-regulated	Fra-1 proto-oncogene	inducing macrophage polarization
miR-26b	down-regulated	down-regulated	SLC7A11	apoptosis
miR-29a	down-regulated	up-regulated	Col4a2, Spry1, Timp3	antiangiogenic
miR-30a	down-regulated	down-regulated	MTDH	angiogenesis
miR-30c	down-regulated	down-regulated	KRAS	signalling
miR-125a	down-regulated	down-regulated	HER2, HER3	epidermal growth factor receptors
miR-125b	down-regulated	down-regulated	HER2, HER3	epidermal growth factor receptors
miR-148a	down-regulated	down-regulated	ERBB3	growth factor
miR-148b	down-regulated	down-regulated	ITGA5, ROCK1, PIK3CA, NRAS, CSF1	
miR-181a	down-regulated	up-regulated	ATM	stress-sensor kinase
miR-195	down-regulated	down-regulated	CCNE1	cyclin
miR-196a	down-regulated	up-regulated	ANXA1	apoptosis
miR-320	down-regulated	down-regulated	TRPC5, NFATC3	
miR-374a	down-regulated	up-regulated	WIF1, PTEN, WNT5A	negative regulators of the Wnt/β-catenin signalling cascade
miR-494	up-regulated	up-regulated	PTEN	negative regulator of the Akt/PKB signalling pathway

The discrepancies in the expression levels of miR-29a, miR-181a, miR-196a and miR-374a between dogs and humans may be caused by other target genes having a stronger influence on canine mammary cancer than human breast cancer development, i.e. other genes than those reported in the literature about breast cancer [34, 40, 42, 44]. We found target genes for miR-29a, miR-181a and miR-374a in the dogs’ mammary cancer. The results are included in Table 5.

**Table 5:** Predicted targets for microRNAs differently deregulated in canine mammary cancer from human breast cancer

microRNA	Metastatic canine mammary cancer	Target gene	Metastatic canine mammary cancer	Gene’s name	Gene’s function
cfa-miR-29a	down-regulated	PARD3B	up-regulated	Par-3 family cell polarity regulator beta	cell cycle regulation
cfa-miR-181a	down-regulated	RAD21	up-regulated	RAD21 homolog (S. pombe)	cell cycle regulation, DNA-damage repair, cell differentiation
		BCLAF1	up-regulated	BCL2-associated transcription factor 1	cell differentiation
		PRKAA1	up-regulated	protein kinase, AMP-activated, alpha 1 catalytic subunit	cell differentiation
		YWHAG	up-regulated	tyrosine 3-monooxygenase/tryptophan 5-monooxygenase activation protein, gamma polypeptide	cell differentiation
		ESCO2	up-regulated	establishment of sister chromatid cohesion N-acetyltransferase 2	cell cycle regulation, DNA-damage repair
		LBR	up-regulated	lamin B receptor	receptor, cell adhesion
cfa-miR-374a	down-regulated	SPIN1	up-regulated	spindlin 1	cell cycle regulation
		BUB3	up-regulated	BUB3 mitotic checkpoint protein	cell cycle regulation, cell cycle checkpoint
		RAD21	up-regulated	RAD21 homolog (S. pombe)	cell differentiation, cell cycle regulation, DNA-damage repair
		SKIN	up-regulated	SKI-like oncogene	cell differentiation

Up-regulation of the target genes for these microRNAs leads to a derangement of cell cycle control and cell differentiation and, as a consequence, plays a role in the onset of cancer and, subsequently, in the metastatic progression [27]. However, the differences in the expression profiles of miR-29a, miR-181a, miR-196a and miR-374a may be due to the presence of normal mammary stromal cells in the examined tumour samples, what is sometimes difficult to avoid. Stroma of the normal human mammary gland includes myofibroblasts [46] and that of the dog does not include them [47], so this distinction may be a reason for the microRNA discrepancies between dogs and humans. A role of these microRNAs in myofibroblast differentiation was previously described [48–50].

### Validation of selected microRNAs levels in plasma samples as cancer markers

While deregulated microRNAs in tissue samples regulate known targets involved in tumour development and metastasis, circulating microRNAs might play a role as stable, specific biomarkers for cancer diagnosis and prognosis [51]. Interestingly, Chen et al. showed that the unique signature of microRNAs in blood samples might follow the same pattern as in tumours [52]. However, this trend has not been observed in our study. The PCR analysis showed no significant differences in the expression of selected miRNAs between the metastatic and non-metastatic group. Such dissimilarity between the obtained results from tumour and plasma samples can be challenging to elucidate, as the mechanism of microRNA release into the blood is not fully understood yet. Steudemann et al. pointed out that microRNAs might be released not only by pathologically changed tissue, but also by other organs in the body modifying its final expression in blood samples [53]. Moreover, a recently discovered role of haemolysis in altering plasma levels of microRNAs put an ongoing question regarding other factors with a similar impact [54].

Many factors may play a role in the analysis of plasma microRNA levels. Due to the variety of conditions and used techniques, the process of raw data normalization might be critical to obtain reliable results. To date, there is no endogenous control for the evaluation of circulating microRNAs. An ideal candidate should remain stable in both healthy and affected individuals and resistant to external factors. Several studies proposed miR-16 as a potential housekeeping gene in human studies. However, the expression of this microRNA was down-regulated in the malignant group in our microarray data and therefore it could not be applicable for the evaluation of plasma samples [55, 56]. As a result, we used the expression of a synthetic RNA spike-in (UniSp6) for the internal normalization of microRNA level. The average Ct values within groups were detectable in all the investigated samples, however, did not show any differences between the groups.

## Conclusions

In summary, the microRNA profiling of canine mammary cancer has identified microRNAs that are differentially expressed according to the tumour type, malignancy grade and metastasis factor regardless of the tumours’ histological type. The most significant difference in microRNA expression has been found between the metastatic and non-metastatic group. These results are very interesting because, firstly, they suggest that microRNAs regulate mostly the metastasis process (not the malignant transformation) and, secondly, they may constitute molecular markers of metastasis. This is of great predictive importance for the course of a disease because some histopathologically identical malignant tumours have a different clinical outcome. Moreover, due to the microRNA profile similarities between canine mammary cancer and human breast cancer, metastasis biomarkers for dogs can also be further examined as useful for humans.

## Additional files

Additional file 1: Target sequences for validation primer sets – microarray results. (XLSX 43 kb)

Additional file 2: Primers’ sequences for selected targets of microRNAs deregulated in canine mammary cancer. (XLSX 46 kb)

Additional file 3: Characteristics of the plasma samples. Legend: x − the factor does not concern the sample, − − the malignancy grade factor does not concern the sample (for benign tumours), [] – samples derived from the same dogs which tumours were used for microarray analysis. (XLS 42 kb)

Additional file 4: Target sequences for validation primer sets – plasma. (XLSX 41 kb)

Additional file 5: Characteristics of the tumour samples. Legend: x − the factor does not concern the sample, − − the malignancy grade factor does not concern the sample (for benign tumours), empty spaces − data not available. (XLSX 32 kb)

Additional file 6: Differentially expressed microRNAs for the tumour type factor. Significance Analysis of Microarrays (SAM) test and one-way ANOVA test. Legend: avg. Hy3 − average array signal intensity of the probes, d(i) − observed relative difference, (Ctrl) − control, (Benign) − benign tumour, (Mal) − malignant tumour, FDR - false discovery rate. (XLSX 62 kb)

Additional file 7: Differentially expressed microRNAs for the malignancy grade factor. Significance Analysis of Microarrays (SAM) test and one-way ANOVA test. Legend: avg. Hy3 − average array signal intensity of the probes, d(i) − observed relative difference, FDR − false discovery rate. (XLSX 63 kb)

Additional file 8: Differentially expressed microRNAs for the metastasis factor. Significance Analysis of Microarrays (SAM) test and one-way ANOVA test. Legend: avg. Hy3 − average array signal intensity of the probes, d(i) − observed relative difference, (No) − non-metastatic tumour, (Yes) − metastatic tumour, FDR − false discovery rate. (XLSX 69 kb)

Additional file 9: Predicted targets for microRNAs deregulated in metastatic canine mammary cancer. (XLSX 21 kb)

## Abbreviations

FRAs: Fragile sites
HOXD10: homeobox D10
miRNA: microRNA
PBS: Phosphate buffered saline
PCA: Principal Component Analysis
RT-qPCR: Real-time quantitative PCR
SAM: Significance Analysis of Microarrays
VEGF: Vascular endothelial growth factor
WHO: World Health Organization
